# Secondary traumatic stress and vicarious posttraumatic growth in oncology nurses: the mediating role of empathy

**DOI:** 10.3389/fpubh.2024.1454998

**Published:** 2024-09-17

**Authors:** Yitong Cai, Ming Liu, Yifei Li, Juan Li, Jie Geng, Xiaoying Liu, Jingping Zhang

**Affiliations:** ^1^Xiangya School of Nursing, Central South University, Changsha, Hunan, China; ^2^Evidence-Based Medicine Center, Lanzhou University, Lanzhou, Gansu, China; ^3^Department of Neurosurgery, Lanzhou University Second Hospital, Lanzhou, Gansu, China; ^4^Department of Gynecologic Oncology, Fourth Hospital of Hebei Medical University, Shijiazhuang, Hebei, China

**Keywords:** secondary traumatic stress, vicarious posttraumatic growth, oncology nurses, empathy, mediating role

## Abstract

**Background:**

The relationship between secondary traumatic stress (STS), empathy, and vicarious post-traumatic growth (VPTG) in oncology nurses is unclear. Understanding these links is crucial for improving nurse well-being and patient care quality.

**Objective:**

This study aimed to investigate the relationships between STS, empathy, and VPTG among oncology nurses.

**Methods:**

This cross-sectional study was conducted in a multicentric setting. Data collection involved oncology nurses completing questionnaires assessing STS, empathy, and VPTG levels. Data analysis included correlation analyses, multiple stepwise regression analysis, and structural equation modeling (SEM) to examine the interrelationships between these variables. The study adhered to the STROBE checklist.

**Results:**

A total of 391 oncology nurses participated in the study. They showed moderate to low levels of VPTG and high levels of STS. STS exhibited a negative association with VPTG, while empathy demonstrated a positive direct association with both VPTG and STS. Moreover, SEM indicated that empathy mediated the relationship between STS and VPTG, with a partial mediating effect of 0.127. Factors such as receiving psychological training, educational attainment, STS, and empathy collectively explained 24% of the variance in VPTG.

**Conclusion:**

Our findings highlighted the negative correlation between STS and VPTG among oncology nurses. Additionally, empathy was found to mediate the relationship between STS and VPTG, suggesting it plays a significant role in influencing VPTG.

**Implications for practice:**

To aid oncology nurses, interventions should focus on reducing STS and enhancing empathy. Strategies like resilience workshops, peer support, and stress management can foster VPTG. Creating a supportive work environment is crucial for nurses’ well-being and quality patient care.

## Introduction

Cancer prevention and treatment pose significant challenges and burden on public health, making the development of cancer medical nursing services a crucial task in China healthcare system (1, 2). High-quality oncology care services necessitate both professional nursing skills and ample emotional support for patients and their families (3, 4). Nurses in clinical oncology settings frequently endure prolonged periods of significant stress due to factors like the complex and variable nature of patients’ conditions, frequent unexpected events, and the need for extended working hours (5). Moreover, the high level of specialization and lengthy duration of oncology treatment foster a long-term and close relationship between nurses and patients (6, 7). During this prolonged nurse–patient interaction, patients’ suffering, negative emotions, and unfortunate experiences also affect nurses’ emotional well-being and cognition (8, 9).

Increasing research indicates that secondary traumatic stress (STS) is common among nursing professionals, with oncology nursing being particularly susceptible, the distinct traumatic encounters faced by oncology nurses increase their vulnerability to developing STS (8, 10, 11). STS refers to the subsequent behaviors and emotional responses that naturally arise from indirect exposure to others’ traumatic events, often stemming from assisting or desiring to assist individuals experiencing trauma or distress (12). Estimates suggest that the prevalence of STS among oncology nurses ranges from 38 to 60%, higher than in other general departments (13, 14). Nurses with STS may exhibit physiological symptoms including elevated heart rate, breathing difficulties, and sleep disruptions, additionally, this condition can have adverse effects on nurses and their professional performance, leading to reduced achievement, increased staff turnover rates, and negative emotional states. These outcomes significantly impact both the quality of work and life, as well as the overall quality of nursing care and management (15, 16).

Beyond the negative impacts, in recent years, researchers have begun to focus on growth resulting from indirect trauma. Some studies suggest the possibility of positive changes as well (16, 17). Calhoun defined the personal growth and meaning gained through others’ trauma as vicarious posttraumatic growth (VPTG) (9). Similar to posttraumatic growth (PTG), VPTG manifests as positive changes in self-awareness, interpersonal relationships, and life perspectives for individuals (8). While VPTG shares similarities with Similar to posttraumatic growth, there are also some distinctions (12). Posttraumatic growth is a form of growth that arises directly from experiencing trauma, while VPTG is a form of growth that arises indirectly from experiencing trauma. These two phenomena are fundamentally distinct (10, 18–20). Promoting nurses’ VPTG effectively alleviates the stress and anxiety caused by exposure to indirect traumatic events in their work, maintaining their mental well-being, enhancing job satisfaction, thereby boosting their dedication and positivity at work, ultimately improving work efficiency and quality (21, 22).

Research has shown that there is a certain degree of VPTG among the nursing population (23–25). In medical settings, trauma exposure is typically indirect. Therefore, the association between trauma symptoms and growth often takes the form of the STS-VPTG link (25). Many studies have confirmed the correlation between STS and VPTG, but the conclusions vary. Some studies suggest a positive correlation between STS and VPTG, indicating that experiencing STS can lead to personal growth through adaptive coping and emotional processing, conversely, other studies suggest a negative correlation, where high levels of STS inhibit the development of VPTG due to overwhelming stress and maladaptive coping mechanisms (25–27). The inconsistency in these findings may be due to several factors, including differences in sample characteristics like population demographics (age, gender, professional background), different contexts and severities of trauma exposure in various medical settings, and the use of diverse measurement tools to assess STS and VPTG, some of which may not be sensitive enough to capture the true nature of the relationship between these variables, leading to errors and misinterpretations. Controlling for demographic data, different contexts and severities of trauma exposure in medical settings, and using consistent measurement tools, we found only four studies that investigated the relationship between STS and VPTG in the nursing population. Among these, three studies confirmed a negative correlation between STS and VPTG (26, 28, 29). Although one study found a positive correlation, its sample included nurses with less than 1 year of clinical experience, which differs significantly from the sample in this study. Therefore, this study hypothesizes a negative correlation between VPTG and STS.

Empathy, as defined, involves therapists’ comprehension and experiential connection with patients’ emotions and thoughts, which is then demonstrated through expressions of concern, warmth, and respect (30). Nurses’ empathy represents their emotional acuity within clinical settings, enabling them to accurately discern both their own and patients’ emotions, thereby facilitating better patient understanding, addressing patients’ physical needs, and alleviating their psychological distress (31). A dearth of empathy among nurses can lead to significant repercussions for patients (32). Comparatively, oncology patients exhibit heightened empathetic care requirements in contrast to other departments such as Emergency and ICU (33). Research suggests that nurses with elevated empathy levels are more adept at comprehending patient emotions, fostering harmonious nurse–patient relationships, bolstering personal fulfillment, and mitigating professional burnout (34).

Studies further demonstrate the positive impact of nurses’ empathy on VPTG. A meta-analysis, incorporating a qualitative inquiry into VPTG, synthesizes a model underscoring the pivotal role of empathy in VPTG occurrence (27, 35). Furthermore, the study validated that empathy is significantly related to the development of STS among oncology nurses exposed to traumatic events (36). The integrated review conducted by Sheen et al. revealed that empathy is consistently related to STS among emergency department nurses across both quantitative and qualitative literature (37, 38). In his book, Figley discusses compassion fatigue and STS, noting that prolonged exposure to STS may lead to an influence in caregivers’ empathy, as they may become emotionally numb as a self-protection mechanism (7). Based on the conclusions from the preliminary literature review and the negative correlation between STS and VPTG, we hypothesize that empathy mediates the relationship between STS and VPTG among nurses.

Current research confirms the correlation among the three factors, yet the existing studies suffer from the following limitations: (1) Recent studies by overseas scholars have employed various methodologies to address individuals at heightened risk of STS. However, most scholarly investigations primarily rely on assessment tools such as the Professional Quality of Life (ProQOL) scale and the Compassion Fatigue Self-Test (CFST) to evaluate STS. Conversely, the use of the Secondary Traumatic Stress Scale (STSS), designed to measure the intensity of anxiety, depression, or PTSD symptoms associated with STS, remains notably limited. The precise assessment, diagnosis, and management of STS are essential (17). (2) Current studies often fail to differentiate the impact of personal trauma history, direct trauma, and exposure to indirect trauma on growth experiences when utilizing the PTGI to assess VPTG among nurses. This direct application of the PTGI compromises the accuracy of measurement results, thus offering less precise influencing factors and predictive variables of VPTG (39). (3) There has been no exploration of the mediating role of empathy in the relationship between STS and VPTG, particularly among oncology nurses.

Therefore, this study aimed to explore the mediating role of empathy between STS and VPTG among oncology nurses using a profession-specific survey instrument. Additionally, it investigates the current status of empathy, STS and VPTG among oncology nurses, identifies influencing factors of VPTG.

The study proposes the following hypotheses:

Hypothesis 1: STS negatively predicts the VPTG of oncology nurses.

Hypothesis 2: Empathy is positively correlated with VPTG.

Hypothesis 3: Empathy mediates the negative relationship between STS and VPTG among oncology nurses.

## Method

### Design

This study adopted a cross-sectional design, and in order to uphold research rigor, the findings were reported utilizing the STROBE checklist (40).

### Participants

For the sake of sample diversity and research feasibility, this study employed convenience sampling. From September to November 2023, three hospitals were selected in three cities (Henan, Gansu, and Hunan) in central and western China. The participants were nurses who met the following inclusion criteria: (1) employed in oncology departments; (2) holding valid nursing licenses; (3) voluntary participation with informed consent. Nurses who did not meet the following exclusion criteria were excluded: (1) those not on duty during the survey period, including those on sick leave, vacation, or participating in continuing education programs; (2) those who had been off duty for more than 6 months or on maternity leave during the survey period.

### Data collection

This study employed an online survey method. Researchers designed an electronic version of the survey questionnaire using the WenJuanXing platform.^1^ After obtaining informed consent, participants voluntarily completed and submitted the questionnaire. To ensure anonymity, all data were collected and stored anonymously in a secure database, accessible only to the research team.

### Sample size

Sample calculation is based on two principles: (1) A minimum sample size of 200 is required for structural modeling (41), (2) calculated as 5–10 times the number of scale items, 115–230 responses are needed (16). Considering a 20% rate of invalid questionnaires, this study requires a minimum sample of 240.

### Instruments

#### Questionnaire for general information

The researchers designed a survey questionnaire based on the purpose and content of this study, referencing relevant literature. This questionnaire includes basic information such as gender, age, education level, years of work experience, and department affiliation.

#### The secondary traumatic stress scale

The Secondary Traumatic Stress Scale (STSS) was utilized to assess the level of STS. Originally developed by Bride et al. (42), the scale was subsequently translated into Chinese by Li et al. (43) The STSS comprises 17 items assessing the intensity of STS experienced in the preceding 7 days. Responses are recorded on a 5-point scale, ranging from 1 to 5. Total scores falling below 28 suggest minimal or negligible STS, scores ranging from 28 to 37 indicate mild STS, scores between 38 and 43 suggest moderate STS, scores from 44 to 48 indicate severe STS, while scores of 49 or above indicate extreme STS. The scale demonstrated a Cronbach’s α of 0.971.

#### The vicarious posttraumatic growth inventory

The Vicarious Posttraumatic Growth Inventory (VPTGI) was utilized to assess the level of VPTG. Originally developed by Deaton (39), the inventory was subsequently translated into Chinese by our team with authorization from Deaton (39). The Chinese version of the VPTGI comprises four dimensions with a total of 23 items, demonstrating satisfactory reliability and validity. Responses are recorded on a 5-point scale, ranging from 1 to 5. Based on the total score, scores below 100 indicate the low-score group, while scores above 118 indicate the high-score group. The inventory exhibited a Cronbach’s *α* of 0.970.

#### The interpersonal reactivity index

This study utilized the Chinese version of the Interpersonal Reactivity Index (IRI-C), which was compiled by Davis, translated by Zhan Zhiyu, and revised by Zhang Fengfeng, to assess the empathy levels of nurses (44, 45). The questionnaire has been widely used to measure empathy among Chinese nurses, and various studies have confirmed its good reliability and validity (35, 46, 47). The scale consists of 22 items, employing a Likert 5-point rating scale ranging from 0 (not appropriate) to 4 (very appropriate). The theoretical midpoint of the scale is 44 points, with an individual item’s theoretical midpoint being 2 points. The total score ranges from 0 to 88 points, with higher scores indicating stronger levels of empathy in individuals. The Cronbach’s alpha coefficient for the total scale was 0.754.

### Statistical analysis

Data analysis was performed using IBM SPSS Statistics and AMOS (version 25.0; IBM, Chicago, IL, USA). Descriptive statistics, such as means, standard deviations, frequencies, and percentages, were employed to analyze demographic data, empathy, VPTG, and STS. Pearson’s correlation analyses were conducted to explore the relationships between variables, while ANOVA and t-tests were utilized to examine sociodemographic differences in STS, empathy, and VPTG. Multiple linear regression analysis was conducted to ascertain the impact of variables on VPTG. All statistical tests were two-tailed (*α* = 0.05).

A structural equation model (SEM) was employed to investigate the correlation among STS, empathy, and VPTG among oncology nurses, and to assess the mediating role of empathy. I Model fit was evaluated using various indices including *χ*^2^/df, Tacker-Lewis index (TLI), comparative fit index (CFI), incremental fit index (IFI), relative fit index (RFI), normal fit index (NFI), and root mean square error of approximation (RMSEA). A *χ*^2^/df value less than 3 indicates a good fit with the observed data. TLI, CFI, IFI, RFI, and NFI values above 0.90 reflect a well-fitting model. An RMSEA value of 0.08 or lower signifies an acceptable level of approximation error, supporting the model’s fit (48). Additionally, statistical significance was set at *p* < 0.05 (two-tailed test).

### Ethical considerations

This study received approval from the Institutional Review Board at Central South University (approval number: E2023110). Before initiating the survey, the principal investigator liaised with the nursing department of the surveyed hospital to elucidate the study’s objectives and nature, securing endorsement from the hospital, nursing department, and pertinent units.

## Results

### Sample characteristics

We distributed 400 questionnaires. Of these, six respondents opted not to complete them, and an additional three were excluded due to insufficient completion time. Ultimately, 391 valid questionnaires were included. The majority of participants were female (*n* = 381), and there were 244 individuals aged between 30 and 40 years old. The mean age of the participants is 34.93 ± 17.584. The highest proportion of nurses had 11–20 years of professional experience. The majority of nurses held a bachelor’s degree (*n* = 351). The numbers of nurses who had received psychological training and those who had not were approximately equal. Participants encompassed a wide range of specialty departments. Further sociodemographic characteristics of the nurses are delineated in Table 1.

**Table 1:** Demographic characteristics and scores of VPTG, STS and empathy.

Variables	Category	*n*	VPTG				STS				Empathy			
			Mean	SD	F/t	*p*	Mean	SD	F/t	*p*	Mean	SD	F/t	*p*
Gender														
	Male	10	108.5	11.93	−0.2	0.85	49.5	16.15	0.83	0.41	50.3	6.13	−0.4	0.69
	Female	381	109.5	15.46			45.64	14.5			51.55	9.86		
Age (years)														
	20–30	101	107.72	15.38	4.27	**0.01**	46.28	14.77	1.91	0.13	49.27	9.8	3.64	**0.01**
	31–40	244	108.74	15.98			46.04	14.13			51.85	9.26		
	41–50	39	117.26	15.33			44.69	15.64			54.36	11.95		
	>50	7	117	18.74			33.14	15.79			56.43	8.02		
Professional title														
	Nurse	29	112.24	17.07	0.93	0.45	44.31	14.22	2.36	0.053	49.24	11.04	0.59	0.671
	Nurse Practitioner	112	108.98	15.56			46.36	15.25			51.18	9.61		
	Nurse in Charge	217	108.73	15.98			46.31	13.64			51.82	9.71		
	Associate Chief Nurse	28	113.39	17.02			43.46	17.71			52.71	9.38		
	Chief Nurse	5	115	15.75			27.6	7.37			52.2	12.74		
Department														
	Internal Medicine	326	108.86	16.38	0.78	0.58	45.72	14.14	1.44	0.20	51.05	9.79	1.06	0.38
	Oncology Surgery	17	114.29	12.49			44.59	15.97			53.59	9.72		
	Radiation Oncology	15	108.8	14.13			46.3	16.14			51.9	7.96		
	Traditional Chinese Medicine and Western Medicine	8	116.4	17.76			55.8	23.91			57.4	10.32		
	Interventional Radiology	10	114	11.12			39.6	12.38			54.8	13.64		
	Hematology	8	113	14.91			39.6	12.03			52.9	7.47		
	Miscellaneous	7	109.4	15.09			52.4	15.28			54.6	6.9		
Years of nursing experience														
	<1	4	114	6.48	2.42	**0.03**	46	20.31	3.36	**0.00**	53.25	5.74	2.8	**0.01**
	1–2	12	101.92	12.36			54.25	13.15			49.58	8.32		
	3–5	35	106.26	13.73			43.06	13.27			47.74	10.17		
	6–10	103	109.91	16.18			44.49	14.02			50.39	9.69		
	11–20	198	108.71	16.13			46.55	14.48			51.94	9.42		
	21–30	33	116	16.7			48.15	15.34			56.15	11.03		
	>30	6	122.17	15.61			25.33	6.5			56.17	8.75		
Education level														
	Diploma	27	120.04	17.48	6.72	**0.00**	49.74	16.05	1.11	0.33	54.15	8.98	1.21	0.30
	Bachelor degree	351	108.59	15.78			45.46	14.34			51.38	9.91		
	Master degree or above	13	111.54	11.35			44.85	16.38			49.85	6.84		
Marital status														
	Married	307	110.2	15.93	2.88	0.06	45.1	14.19	1.9	0.15	52.3	9.46	8.75	**0.00**
	Unmarried	77	106	16.36			48.5	15.22			47.8	10.15		
	Divorce	7	116.9	9.12			41.6	19.77			58.6	8.72		
Frequently engage in end-of-life care for patients														
	Yes	224	109.6	17.1	0.16	0.87	45.3	15.01	−0.72	0.47	50.8	10	−1.67	0.1
	No	167	109.3	14.47			46.3	13.88			52.5	9.41		
Received training and education on psychological trauma courses														
	Yes	190	112.53	15.55	3.72	**0.00**	45.05	14.56	−0.9	0.37	52.23	9.78	1.41	0.16
	No	201	106.59	15.94			46.38	14.51			50.84	9.75		

VPTG, vicarious posttraumatic growth; STS, secondary traumatic stress. Bold values indicate statistically significant differences.

### Univariate analyses of the factors associated with secondary traumatic stress, vicarious posttraumatic growth and empathy

Through ANOVA analysis and t-tests, it was found that age, years of nursing experience, education level, and receipt of training on psychological trauma courses influenced VPTG. In our survey, only years of nursing experience impacted STS, while age, years of nursing experience, and marital status affected empathy. Please refer to Table 1 for detailed information.

### Secondary traumatic stress, vicarious posttraumatic growth, empathy and their associations

The mean ± standard deviation of the total score for VPTG was 109.48 ± 16.01, indicating a moderate overall level. Specifically, 76% of nurses scored at a moderate to low level of VPTG. The mean ± standard deviation of the total score for STS was 45.73 ± 14.53, suggesting a severe level, with 47.1% of nurses scoring at an extreme level of STS. The mean ± standard deviation of empathy was 51.52 ± 9.78. Specific details can be found in Table 2.

**Table 2:** Mean and standard deviations of variables.

Variables	Mean	SD	Frequency	Percentage
VPTG	109.48	16.01		
Low level			127	32.5
Average levels			170	43.5
High level			94	24.0
STS	45.73	14.53		
None			42	10.7
Mild			82	21.0
Moderate			43	11.0
Severe			40	10.2
Extreme			184	47.1
Empathy	51.52	9.78		

VPTG, vicarious posttraumatic growth; STS, secondary traumatic stress.

The correlation between VPTG, STS, and empathy is presented in Table 3. STS demonstrates a negative correlation with VPTG and its various dimensions, while showing a positive correlation with empathy. Empathy exhibits a positive correlation with VPTG and its various dimensions.

**Table 3:** Correlations of STS, Empathy, and VPTG.

	VPTG	Personal growth and care	Professional balance and inspiration	Professional meaning and self-awareness	Development and maintenance of personal relationships	Empathy
Personal growth and care	0.933**					
Professional balance and inspiration	0.928**	0.783**				
Professional meaning and self-awareness	0.904**	0.789**	0.792**			
Development and maintenance of personal relationships	0.725**	0.596**	0.620**	0.641**		
Empathy	0.359**	0.299**	0.385**	0.295**	0.281**	
STS	−0.187**	−0.182**	−0.125*	−0.243**	−0.119*	0.275**

**p < 0.01; *p* < 0.05; VPTG, vicarious posttraumatic growth; STS, secondary traumatic stress.

### Regression analyses examining covariates of vicarious posttraumatic growth

This study employs nurses’ VPTG scores as the dependent variable and selects general data showing significant differences (*p* < 0.05) in univariate analysis, along with factors related to VPTG, as independent variables. Subsequently, multiple regression models are established and subjected to multivariate regression analysis. As illustrated in Table 4, the variance inflation factors (VIFs) of all independent variables fall between 1 and 3, indicating the absence of significant multicollinearity. The analysis suggests that low levels of STS, high empathy, high educational level, and psychological training are the primary predictive factors for a high level of VPTG (*F* = 31.715, *R*^2^ = 0.24, *p* < 0.05). This implies that 24% of the variance in VPTG is explained by STS, empathy, educational level, and psychological training.

**Table 4:** Multiple linear regression analysis examining covariates of VPTG (*n* = 391).

Outcome variables	Explanatory variables	*B*	Beta	*t*	*p*	VIF	*F*	Adjusted *R*^2^
VPTG	Empathy	0.698	0.426	9.230	0.000	1.093	31.716	0.24
	STS	−0.336	−0.305	−6.613	0.000	1.090		
	Training and education^*^	−4.138	−0.129	−2.896	0.004	1.023		
	Education level	−5.166	−0.103	−2.301	0.022	1.022		

* Received training and education on psychological trauma courses.

VPTG, vicarious posttraumatic growth; STS, secondary traumatic stress.

### The mediating role of compassion satisfaction on the relationships between secondary traumatic stress and vicarious posttraumatic growth

**Figure 1:**
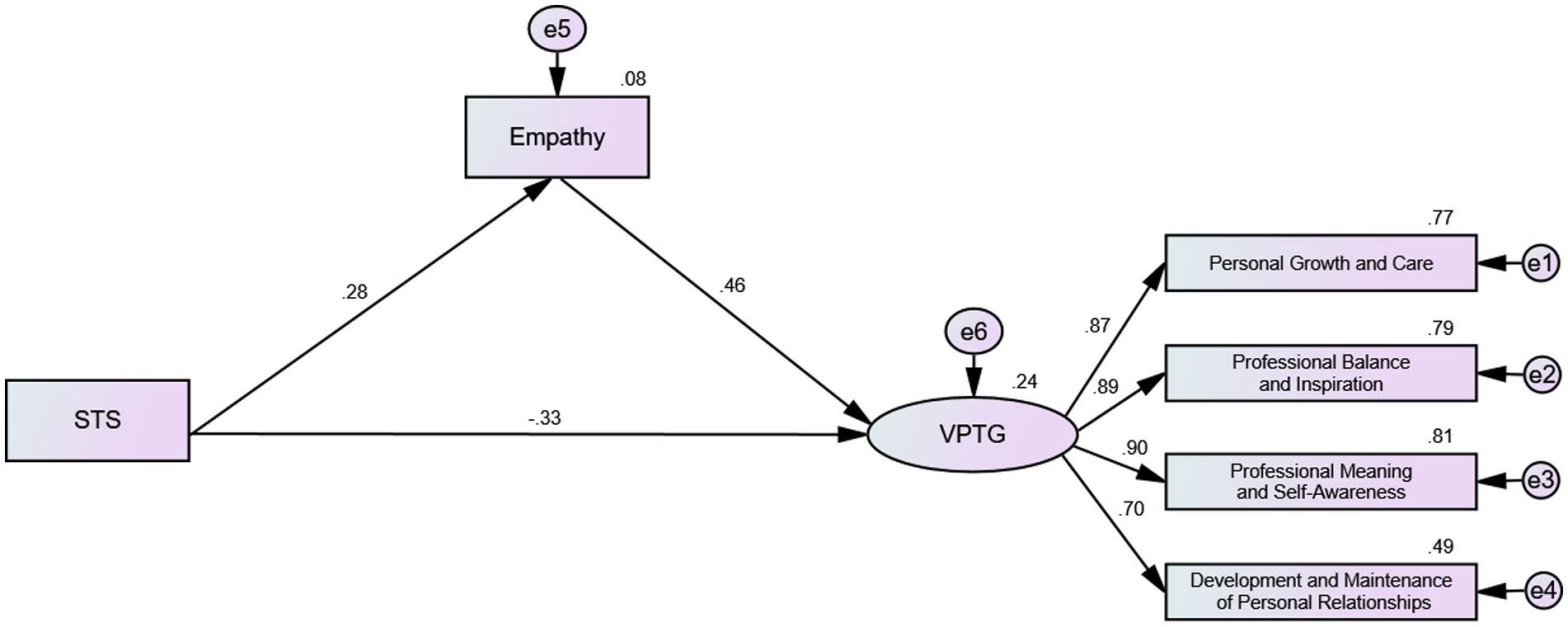
The Mediate Effect Model of Empathy Between STS and VPTG.

The structural equation modeling technique was employed to examine the hypothetical model, which encompassed three latent constructs (STS, empathy, and VPTG) and four observed variables (Figure 1). The model demonstrated satisfactory fit, as evidenced by the following indices: *χ*^2^/df = 2.721, TLI = 0.979, CFI = 0.989, IFI = 0.989, RFI = 0.967, NFI = 0.982, RMSEA = 0.066. Furthermore, all factor loadings of indicators on latent constructs were statistically significant (*p* < 0.05), indicating adequate representation of all latent constructs by their respective indicators. The mediating role of empathy was evaluated through 5,000 bootstrap analyses with 95% confidence intervals. As illustrated in Table 5, STS exerted a significant direct effect on both empathy (*β* = 0.275, *p* = 0.000) and VPTG (*β* = −0.333, *p* = 0.000). The direct effect of empathy on VPTG was determined to be 0.462 (*p* = 0.000). Moreover, the indirect effect of STS → empathy → VPTG was estimated to be 0.127 (*p* = 0.000), suggesting partial mediation of the relationship between STS and VPTG by empathy.

**Table 5:** Direct and indirect effects for the model (*N* = 391).

Model pathways	Standardized effect (*β*)	95% CI	*p*
Total effect	0.462	(0.363, 0.550)	0.001
Direct effect			
STS → Empathy	0.275	(0.174, 0.375)	***
Empathy → VPTG	0.462	(0.362, 0.551)	***
STS → VPTG	−0.333	(−0.434, −0.232)	***
Indirect effect			
STS → Empathy → VPTG	0.127	(0.076, 0.189)	***

***p < 0.001; VPTG, vicarious posttraumatic growth; STS, secondary traumatic stress.

## Discussion

In this study, we revealed the levels of empathy, STS and VPTG among oncology nurses in certain regions. Additionally, we explored the mediating role of empathy and investigated factors that may influence VPTG. The results indicated that oncology nurses exhibited above-average levels of empathy, severe levels of STS, and moderate levels of VPTG. These findings suggest that oncology nurses commonly experience significant emotional burdens due to indirect trauma, but also demonstrate some positive changes. Empathy plays a mediating role in mitigating the negative impact of STS on VPTG. These results underscore the importance of managing and reducing STS among nurses through psychological training and support systems, while enhancing their empathy and VPTG.

Our findings indicate that oncology nurses scored 51.53 ± 9.78 in empathy, indicating an above-average level. This conclusion surpasses the cross-sectional survey results of nurses in the Hunan region of China and exceeds findings from another study assessing the empathy status among nursing students (49). The discrepancy in these outcomes may stem from differences in study populations. The specialized nature of oncology nursing duties entails a heightened requirement for empathetic care, which could account for the observed differences in research outcomes (50). The STS score of oncology nurses was 45.73 ± 14.53, indicating severe STS. Among them, 47.1% of nurses experienced extremely severe STS levels. Two surveys on STS among oncology nurses showed moderate to high levels, despite different assessment tools used. These findings suggest a prevalent occurrence of severe STS among nurses (36, 51). Future attention should be focused on interventions for the STS of nursing professionals. The VPTG score was 109.48 ± 16.01, indicating a moderate level, this outcome was consistent with previous studies (52). Specifically, 76% of nurses had VPTG scores in the mid-to-low range, while only 24% scored higher. This marks the first investigation of VPTG levels among oncology nurses using a specific tool. The study findings suggest that the VPTG levels of oncology nurses after experiencing indirect trauma are generally not high and they commonly experience severe STS. Promoting the reduction of STS and enhancing VPTG is a noteworthy issue deserving attention.

Variations in VPTG and empathy levels exist among oncology nurses of different ages. This could be attributed to the changes in individuals’ experiences, life circumstances, and psychological development as they age, impacting how they perceive and respond to indirect trauma and others’ emotions (53). Additionally, differences in VPTG, STS, and empathy levels among oncology nurses with varying years of work experience suggest a correlation with the development of coping mechanisms and emotional responses over time. With accumulating clinical experience, nurses may become more adept and self-assured in managing diverse diseases and medical conditions (54). Oncology nurses with different educational backgrounds exhibit differing levels of VPTG, Nurses with higher education often receive more extensive professional training, equipping them with broader skills to effectively cope with traumatic events. This enhances their ability to navigate challenges, promoting their post-traumatic growth (VPTG). Additionally, educational background may influence nurses’ resilience and emotional stability, affecting their perception and coping with traumatic events (55). Marital status influences empathy levels among oncology nurses. It may impact nurses’ empathy levels by providing support and understanding from a spouse, potentially enhancing their ability to empathize with patients and colleagues. Moreover, marital stability and satisfaction could contribute to nurses’ emotional well-being, affecting their capacity for empathy (56). Oncology nurses who undergo psychological training exhibit higher levels of VPTG, but there is no significant difference in STS levels. This suggests that psychological training provides nurses with effective coping strategies to manage workplace trauma and promote growth. Understanding the factors influencing VPTG becomes crucial, especially considering that some studies suggest STS is unavoidable. Thus, promoting the transformation of STS into VPTG is paramount (57).

The study supports Hypothesis 1 by demonstrating a negative correlation between STS and VPTG. The findings indicate that higher levels of STS are associated with lower levels of VPTG among oncology nurses. There is a negative correlation between STS and VPTG. This conclusion aligns with previous research findings (26, 28). This suggests that nurses experiencing higher levels of STS may undergo lower levels of VPTG. This phenomenon could be attributed to the overwhelming nature of experiencing indirect trauma, which may hinder individuals’ ability to perceive growth opportunities in the aftermath of trauma. Although there is a negative correlation, a certain level of STS may be necessary for VPTG, indicating a more complex relationship between these variables. This implies that while high levels of STS may impede VPTG due to the intense emotional burden, moderate levels of STS might serve as a catalyst for growth by encouraging deeper emotional processing and resilience-building. High levels of STS can create a significant emotional burden, leading to emotional exhaustion and psychological fatigue among nurses. This exhaustion reduces job satisfaction and professional fulfillment, making it difficult for nurses to find positive meaning and growth opportunities in their traumatic experiences. Additionally, high levels of STS may lead to the development of emotional numbing or depersonalization as psychological defense mechanisms, which can diminish empathy and negatively impact professional performance and psychological well-being. Moderate levels of STS can act as a challenge that stimulates deeper emotional processing and reflection. Nurses facing moderate stress may become more attentive to their emotional responses and psychological needs, prompting them to seek effective coping strategies and support resources. This manageable level of stress can enhance nurses’ resilience and coping abilities, allowing them to discover their inner strength and potential, leading to personal growth and professional development. Additionally, moderate STS fosters empathy, as nurses continually adjust their emotional responses and support strategies when dealing with patients’ distress (58). This adjustment process helps them better understand and respond to patients’ emotional needs, thereby facilitating VPTG. This dual role of STS underscores the importance of managing stress levels among nurses to optimize their professional development and psychological well-being. Understanding this complex relationship is crucial for effective nursing management and mental health interventions, helping to devise more effective professional support and mental health promotion strategies. Furthermore, multiple stepwise regression analysis revealed that empathy, STS, training and education levels, together explained 24% of the variance in VPTG, emphasizing the impact of these factors on VPTG. These findings reaffirm the negative correlation between STS and VPTG, as well as the positive correlation between empathy and VPTG.

We found a positive correlation between empathy and VPTG, supporting our Hypothesis 2. This finding may suggest that nurses experiencing higher job-related stress exhibit heightened empathy toward others’ distress and feelings, possibly due to their increased attention and understanding of others’ emotions and difficulties. This aligns with previous research and supports the notion of a positive correlation between STS and empathy (59, 60). Furthermore, there is a positive correlation between empathy and VPTG. This could be attributed to the fact that nurses with higher levels of empathy may be more attuned to the emotional experiences of others, allowing them to better cope with indirect trauma and facilitating their VPTG. This finding aligns with existing literature supporting the relationship between empathy and VPTG (17, 61).

Structural equation modeling provided additional insights into the relationships among these variables. The results confirmed that empathy mediates the relationship between STS and VPTG, supporting our Hypothesis 3. The model reaffirmed the relationships between STS and empathy, empathy and VPTG, as well as STS and VPTG. Furthermore, it confirmed the mediating role of empathy in the relationship between STS and VPTG. This implies that STS may affect VPTG not only through direct pathways but also through the intermediary mechanism of empathy. Previous research has shown that while STS can lead to emotional exhaustion and negatively impact nurses’ professional performance, it can also prompt nurses to reevaluate their personal values and develop resilience in the face of challenging work environments (62). In this study, the STS experienced by oncology nurses may directly result in lower levels of VPTG. This could be because STS negatively impacts the psychological and emotional states of nurses, hindering their positive growth in response to trauma. Additionally, through the mediating effect of empathy, STS positively influences VPTG. This may occur because the development of empathy helps nurses better understand and respond to the emotional needs of others, thereby facilitating their positive growth in response to trauma. Therefore, despite experiencing STS, nurses may still derive positive psychological growth and development from their traumatic experiences through the development of empathy.

### Limitations

This study also has some limitations. Firstly, as a cross-sectional survey, it relies on participants’ recall and self-reporting, which may introduce recall bias and self-reporting bias, potentially affecting the reliability and accuracy of the study results. Secondly, cross-sectional surveys typically only observe correlations between variables and cannot establish causal relationships. Moreover, while the study focused on STS, empathy, and VPTG, future research should explore additional factors such as the workplace environment, negative life events, and personal coping mechanisms. These factors could provide a more comprehensive understanding of STS and VPTG and help develop better support strategies. Therefore, caution is needed when interpreting the study findings.

### Implications for practice

This study highlights the need for targeted interventions to improve VPTG among oncology nurses, given their high levels of STS and generally moderate to low VPTG. Interventions should focus on managing and reducing STS, as this is crucial for fostering positive growth. While empathy is essential in nursing, “boosting empathy” should be approached cautiously due to its complexity and potential to increase STS. Instead, support programs should balance empathy with self-care strategies, helping nurses manage emotional challenges without heightening STS. By carefully managing STS and supporting empathetic engagement, healthcare organizations can create conditions that promote VPTG, enhancing nurse well-being and patient care. Additionally, future research should explore factors such as the workplace environment, negative life events, and personal coping mechanisms. Understanding these aspects could lead to more comprehensive support strategies, ultimately promoting nurse well-being and improving patient care.

## Conclusion

In conclusion, this study sheds light on the complex interplay between STS, empathy, and VPTG among oncology nurses. The findings highlight the significant impact of STS and empathy on nurses’ psychological well-being and professional growth. Specifically, the study revealed that oncology nurses commonly experience moderate to low levels of VPTG alongside high levels of STS. Importantly, STS was found to have a direct negative effect on VPTG, while also exerting a positive indirect effect through empathy. Additionally, empathy demonstrated a direct positive association with VPTG, suggesting its crucial role in promoting nurses’ VPTG amidst challenging work circumstances.

Overall, by addressing STS and promoting empathy and psychological well-being among oncology nurses, healthcare institutions can create a more supportive work environment conducive to nurses’ personal and professional growth while ensuring the delivery of high-quality patient care. Further research is warranted to explore additional factors influencing VPTG and to evaluate the long-term effectiveness of interventions aimed at promoting nurses’ well-being in oncology settings.

## What is already known about the topic?

- Oncology nurses commonly experience STS.
- Indirect trauma can lead to both STS and VPTG.
- Identifying the factors influencing nurses’ VPTG is essential for exploring interventions to enhance VPTG among nurses.
- Currently, there is a lack of specific assessment tools for measuring VPTG and STS among oncology nurses. This deficiency in measurement tools undermines the validity of related conclusions.

## What this paper adds

- The occurrence of STS among oncology nurses is severe, with VPTG levels being moderate to low. Shedding light on the unique challenges faced by this population.
- The study emphasizes the significant role of empathy in mediating the relationship between STS and VPTG, offering insights into potential pathways for promoting nurses’ well-being and growth.
- By identifying the factors influencing VPTG among oncology nurses, such as the mediating effect of empathy and the impact of STS, the paper provides valuable information for developing targeted interventions and support programs aimed at enhancing nurses’ psychological resilience and promoting their professional development.

## Data Availability

The raw data supporting the conclusions of this article will be made available by the authors, without undue reservation.

## Footnotes

^1^ https://www.wjx.cn/
